# Early Initiation and Exclusivity of Breastfeeding in Rural Zimbabwe: Impact of a Breastfeeding Intervention Delivered by Village Health Workers

**DOI:** 10.1093/cdn/nzy092

**Published:** 2019-02-28

**Authors:** Mduduzi N N Mbuya, Cynthia R Matare, Naume V Tavengwa, Bernard Chasekwa, Robert Ntozini, Florence D Majo, Ancikaria Chigumira, Cynthia M Z Chasokela, Andrew J Prendergast, Lawrence H Moulton, Rebecca J Stoltzfus, Jean H Humphrey, The SHINE Trial Team

**Affiliations:** 1 Zvitambo Institute for Maternal and Child Health Research, Harare, Zimbabwe; 2 Division of Nutritional Sciences, Cornell University, Ithaca, NY; 3 Ministry of Health and Child Care, Harare, Zimbabwe; 4 Blizard Institute, Queen Mary University of London, London, United Kingdom; 5 Department of International Health, Johns Hopkins Bloomberg School of Public Health, Baltimore, MD

**Keywords:** exclusive breastfeeding, early breastfeeding initiation, infants, breast milk, Zimbabwe, village health workers

## Abstract

**Background:**

Suboptimal breastfeeding contributes to >800,000 global child deaths annually. Optimal breastfeeding includes early initiation (EI) and exclusive breastfeeding (EBF) for the first 6 mo.

**Objectives:**

We tested the hypothesis that an intervention targeting context and infant age-specific barriers to EI and EBF will achieve a higher EI and EBF prevalence than those of women participating in the concurrently conducted 2015 Zimbabwe Demographic Health Survey (Z-DHS).

**Methods:**

We designed an intervention to promote EI and EBF, and implemented it within the Sanitation Hygiene Infant Nutrition Efficacy (SHINE) trial in rural Zimbabwe. Intervention modules were delivered at 4 perinatal time points by government-employed village health workers. We compared EI and EBF prevalence among SHINE women who provided outcomes at 1 mo (*n* = 2442) and 3 mo (*n* = 2728), with women in the 2015 Z-DHS.

**Results:**

In cross-sectional analyses EI prevalence was 86.6% and 64.3% in the SHINE and Z-DHS samples, respectively; absolute difference (95% CI) = 22.4% (17.5%, 27.3%). EBF prevalence was similarly high (>80%) in both surveys during the first month of life; during 1 to <2 mo, 2 to <3 mo, 3 to <4 mo, 4 to <5 mo, and 5 to <6 mo, EBF prevalence was, respectively, 85%, 90%, 90%, 84%, and 75% in SHINE, and 71%, 65%, 35%, 26%, and 25% in Z-DHS; absolute difference (95% CI) = 50.2% (34.7%, 65.7%) at 5 to <6 mo. Cesarean delivery, mother's belief that intimate partner violence was sometimes justifiable, and having a male infant negatively modified the effects of the intervention.

**Conclusions:**

The SHINE intervention achieved a high prevalence of EI and EBF. Concurrently addressing gender norms will be critical to make further progress. Formative studies to identify context- and infant age-specific barriers to EI and EBF may inform improvement of breastfeeding practices elsewhere. Important work remains to scale up this intervention beyond a research setting. SHINE was registered at www.clinicaltrials.gov as NCT01824940.

## Introduction

Optimal breastfeeding includes early initiation (EI) within the first hour of delivery; exclusive breastfeeding (EBF) from birth to 6 mo; and continued breastfeeding to at least 24 mo. The

2016 Lancet Breastfeeding Series (1) estimated that achieving very high prevalence of EBF and continued breastfeeding would prevent 823,000 child deaths worldwide annually. An analysis of nearly 100,000 infants from Ghana, India, and Tanzania in the Neovita study recently reported that median (IQR) neonatal mortality rates were 1.41 (1.24–1.62) and 1.79 (1.39–2.30) times greater among infants initiating breastfeeding at 2–23 h and 24–96 h of life, respectively, compared with the first hour (2).

Previous interventions to increase EBF prevalence in Sub-Saharan Africa have included the PROMISE trial which achieved 79%, 82%, and 10% EBF prevalence at 3 mo, and 73%, 50%, and 2% prevalence at 6 mo in, Burkina Faso, Uganda, and South Africa, respectively (3). An EBF intervention among HIV-positive women in South Africa (before antiretroviral therapy during breastfeeding was available) achieved 67% EBF prevalence at 3 mo and 40% prevalence at 6 mo (4).

In Zimbabwe, the 2010 Global Burden of Disease estimated that suboptimal breastfeeding was the leading risk factor for disease burden among children under 5 y (5). The prevalence of HIV among reproductive-aged adults is 15% in Zimbabwe (6), and EBF is the safest mode of infant feeding for HIV-infected mothers in this context (7). Although the Zimbabwe Ministry of Health and Child Care has staunchly promoted breastfeeding over several decades, the prevalence of EI and EBF is not universal. Between the 2010 and 2015 Demographic and Health Surveys (Z-DHS), EBF prevalence did increase, but remained low at infant ages beyond 2 mo: (74%, 46%, and 20% in the 0–1-mo, 2–3-mo, and 4–5-mo groups, respectively), and EI declined from 65% to 58%.

The Zvitambo Institute for Maternal and Child Health Research has been working on EBF promotion in Zimbabwe for the past 2 decades. In our initial urban study population (the ZVITAMBO vitamin A trial, 1997–2001), the EBF prevalence at 3 mo of age was only 7.6%, and virtually no infants were still exclusively breastfed at 6 mo (8). In that trial, EBF, compared with breast milk plus other liquids or foods (i.e., mixed feeding), was associated with a substantially lower risk of breast milk HIV transmission among infants of HIV-positive women. In 2011, we conducted mixed methods formative research to identify and understand the barriers to EBF in rural Zimbabwe (9). We found that ∼80% of infants received nonbreast milk food during the first 2 mo, which was given to prevent or treat an open fontanel or colic. From the second month of life, nonbreast milk foods were given because mothers believed their breast milk was insufficient to satisfy their infant's hunger and thirst. Based on these findings, an intervention was designed for delivery by Zimbabwe Ministry of Health and Child Care village health workers (VHWs) to reach mothers at 4 targeted times: the 7th month of pregnancy, and 1 wk, 1 mo, and 3 mo postpartum. Messages promoted EI and addressed EBF barriers specific to each infant age. The modules, including lesson plans and interactive tools, are publicly accessible at https://osf.io/w93hy.

We implemented this intervention in 2 rural districts of Zimbabwe within the Sanitation Hygiene Infant Nutrition Efficacy (SHINE) trial (10). In this paper, we report EI and EBF prevalence in women who received the intervention through SHINE, and compare them with that of rural mothers and infants in the 2015 Z-DHS. We aimed to assess the effectiveness of the breastfeeding intervention on EI and EBF, and to identify maternal or infant characteristics associated with poor uptake of the promoted behaviors.

## Subjects and methods

The design and methods of the SHINE trial have been previously reported (10). Briefly, SHINE was a cluster-randomized trial testing the effects of improved water and sanitation/hygiene and improved infant and young child feeding; water and sanitation/hygiene + infant and young child feeding together; or standard of care on child stunting and anemia at 18 mo. The study area was the rural population of 2 districts of central Zimbabwe, served by 400 VHWs. Between November 2012 and March 2015, 5280 pregnant women were enrolled at median (IQR) 12 (9, 16) gestational weeks. Women were eligible if they were permanent residents of the rural areas of the study districts, were pregnant, and provided consent. Mothers were tested for HIV by rapid antibody tests. SHINE randomized interventions were delivered during 15 home-visit contacts by VHWs; for ethical reasons, the breastfeeding intervention was delivered by all VHWs to all enrolled women during home visits regardless of study arm. Participant characteristics and trial outcomes, including breastfeeding practices, were assessed by nurses employed by the trial. Given the household nature of the trial, intervention and research visits were only conducted if the mother was available in the household where she consented into the trial. Within the study area, only 1 of the health centers was Baby Friendly certified, but staff in all centers had undergone intensive training on PMTCT, which included a strong EBF component.

The Medical Research Council of Zimbabwe and the Institutional Review Board of the Johns Hopkins Bloomberg School of Public Health reviewed and approved the SHINE trial protocol, including the breastfeeding intervention. Mothers gave written informed consent to participate.

The breastfeeding intervention included 4 modules delivered at 4 time points (Box 1) by VHWs to participating women; each module was about 1 hour in duration, and available family members were invited to participate. Each module built on previous modules, included tools and activities to illustrate key concepts, and used principles of adult learning (11)

**Box 1.** Sanitation Hygiene Infant Nutrition Efficacy study village health worker-led breastfeeding intervention

Module	Target time (allowable range)	Key messages
1	7-mo gestation (33–37 wk)	• Initiate breastfeeding within the first hour of life
		• Feed your infant exclusively with breast milk from birth to 6 mo
		• An open fontanel is normal during infancy; do not give any fluids or traditional herbs in attempt to close the fontanel
2	3 d (0–7 d)	• Correctly position and attach infant to the breast to prevent breast problems and ensure infant receives enough breast milk (village health worker demonstrates and assists mother directly)
3	1 mo (3–8 wk)	• Comfort a colicky infant without feeding nonbreastmilk foods (village health worker demonstrates alternatives such as rocking, burping, and soothing)
4	3 mo (12–16 wk)	• Breast milk is still sufficient to meet your infant's food and water requirements until 6 mo; do not introduce other foods or fluids

VHWs were trained on the breastfeeding intervention over 4.5 d. Didactic instruction provided a foundational understanding of lactation and infant nutrition to prepare VHWs to respond confidently and accurately to a wide scope of questions mothers may ask. Demonstrations focused on the content and flow of the sessions to prepare VHWs to use the module tools, appropriately pace the session, and monitor the mother's understanding. VHWs practiced module delivery through role plays with feedback from their peers. The first time each VHW delivered each module to a SHINE mother, their supervisor prepared them with another role play and then observed the session providing positive feedback and constructive criticism. Continuing supervision was given to VHWs until they demonstrated good performance. VHW supervisors also assessed timing, and overall fidelity of implementation during scheduled visits and spot-checks (12).

Coverage of the intervention modules was high: >80% of women who provided breastfeeding outcomes received all modules, and <1% of mothers who provided outcome data did not receive any module. Accordingly, it was not possible to evaluate the effectiveness of the intervention within the SHINE data set. Therefore, we compared breastfeeding outcomes of women in SHINE with those of rural mothers in the 2015 Z-DHS.

Within SHINE, nurses assessed breastfeeding practices by maternal history during 2 visits conducted at 1 mo (allowable range: 4–12 wk infant age) and 3 mo (12–25 wk infant age). EI was assessed at the 1-mo visit by asking the mother how soon after delivery she first put her infant to the breast; responses ≤1 h were classified as EI. EBF was assessed at the 1-mo and 3-mo visits using a tool previously developed in Zimbabwe (8, 13). Children were classified as EBF if they consumed only breast milk and no other liquids or foods (except vitamins or prescribed medicines) during the previous 24 h.

Within the 2015 Z-DHS, EI was assessed among 1765 rural women who had a child aged <2 y; women were asked how long after delivery they had first put their youngest child to the breast; responses ≤1 h were classified as EI. EBF was assessed among 593 rural women who had an infant <6 mo of age. Children were classified as EBF if they consumed only breast milk and no other liquids or foods during the previous 24 h.

The lists of foods used to assess breastfeeding exclusivity in SHINE and Z-DHS were not exactly alike: traditional medicines and iron-fortified foods were included in the SHINE but not Z-DHS list; insects and organ meats were included in the Z-DHS but not SHINE list. However, no child was classified as mixed fed in either survey solely on the basis of consuming 1 of these 4 groups.

The age distribution differed between the surveys, especially for the first month of life. Most of the SHINE observations for the 0- to <1-mo interval were collected around 28 d, the targeted start of the data collection window for the 1-mo visit, whereas the distribution of infant ages in the 0- to <1-mo interval for the Z-DHS data set was spread evenly across the first 30 d of life. To improve comparability, we included data from SHINE 1-mo assessments that were collected earlier than the targeted window of 4–12 completed weeks; 90 observations collected before 28 d were included.

### Variables and definitions

We define “Early EBF” as EBF practices of infants <12 wk of age, and “Late EBF” as EBF in infants 12–25 wk of age. We estimated risks and risk factors of *not* practicing optimal breastfeeding practices using these terms: “late initiation” (*not* EI); “early mixed feeding” (*not* EBF at 0 < 12 wk), and “late mixed feeding” (*not* EBF at 12–25 wk).

### Statistical analysis

To assess the intervention's effectiveness, we defined intervention exposure in 2 ways: first, enrollment in SHINE (an intent-to-treat approach); and second, receipt of each intervention module. For the second, we created a dichotomous variable for each module, where receipt of the module was coded “yes” and nonreceipt as “no.” By default, all Z-DHS women were assigned a “no” for each module.

We compared the proportions of women practicing EI and EBF by 1-mo infant age groupings across the 2 surveys. Next, we merged the data sets to calculate the unadjusted relative risk (95% CI) of late initiation, early mixed feeding, and late mixed feeding for women in SHINE compared with those in Z-DHS. Finally, using the merged data set, we constructed multivariable Poisson regression models with a log link to estimate the adjusted relative risk (95% CI) for these 3 breastfeeding outcomes for women in SHINE compared with those in Z-DHS and for women who received compared with did not receive each of the 4 modules.

To select the covariates used in multivariable regression analyses, we identified the variables included in both the Z-DHS and SHINE data sets that were plausibly associated with EI, early EBF, and late EBF. We tested these associations within the SHINE set; for associations significant at *P* < 0.2, we tested whether the distribution of the variable differed between the SHINE and Z-DHS data sets to identify potential confounders; variables that were both associated with the outcome and differed across study populations were offered to multivariate models (14). Infant sex, age, and birth weight were included to increase the precision of our estimates. Both Z-DHS and SHINE had previously constructed asset indices as an indicator of wealth. Within each data set, there was no significant association between wealth and breastfeeding outcomes, so a wealth indicator was not included in our models. Mothers’ belief about the acceptability of intimate partner violence was assessed in both surveys (see **Table 1** footnote for specific questions used in each survey).

**TABLE 1:** Maternal, household and infant characteristics of SHINE exclusive breastfeeding data sets at 1 and 3 mo and Z-DHS_2015 data set of rural infants younger than 6 mo^1^

	2015 Z-DHS	SHINE 1-mo data	*P* value vs. 2015 Z-DHS	SHINE 3-mo data	*P* value vs. 2015 Z-DHS
Maternal characteristics					
Age, y, mean ± SD (*n*)	25.6 ± 6.8 (398)	26.77 ± 6.64 (2303)	0.001	26.5 ± 6.7 (2579)	0.014
Height, cm, mean ± SD (*n*)	159.4 ± 6.2 (389)	160.2 ± 5.92 (2352)	0.038	160.2 ± 5.9 (2627)	0.037
Completed schooling, y, median (IQR) (*n*)	9 (7, 11) (398)	10 (9, 11) (2309)	<0.001	10 (9, 11) (2587)	<0.001
Parity median (IQR) (*n*)	2 (1, 4) (398)	2 (1, 3) (1711)	<0.001	2 (1, 3) (2034)	<0.001
Marital status, % married/cohabitating (*n*)	84.2% (335)	95.4% (2189)	<0.001	95.9% (2466)	<0.001
Employment status, % yes (*n*)	18.1% (72)	8.5% (197)	0.027	9.0% (230)	0.031
Believes that being beaten by intimate partner is sometimes justified^2^, % yes (*n*)	40.7% (162)	61.4% (1416)	<0.001	61.5% (1575)	<0.001
HIV status, % positive (*n*)	13.6% (50)	15.8% (382)	<0.001	14.5% (391)	<0.001
Religion, % (*n*)					
Apostolic	54.5% (217)	47.3% (1024)		45.9% (1188)	
Other Christian religion	36.7% (146)	44.3% (1024)	0.015	47.2% (1222)	<0.001
Other religion	8.8% (35)	8.4% (195)		6.9% (179)	
Household characteristics					
Electricity, % yes (*n*)	8.3% (33)	2.5% (57)	0.207	2.7% (68)	0.204
Any latrine, % yes (*n*)	55.3% (220)	39.9% (907)	<0.001	41.6% (1051)	<0.001
Improved floor, % yes (*n*)	51.3% (204)	54.6% (1246)	0.381	55.9% (1414)	0.217
Improved roof, % yes (*n*)	53.7% (200)	11.3% (276)	<0.001	11.3% (305)	<0.001
Time to drinking-water, min, median (IQR) (*n*)	20 (5, 30) (371)	10 (5, 20) (2274)	<0.001	10 (5, 20) (2532)	<0.001
Infant characteristics					
Sex, % female (*n*)	49.0% (195)	49.8% (1216)	0.836	49.6% (1354)	0.876
Infant birth weight, g, mean ± SD (*n*)	3083.6 ± 527.1 (354)	3085.7 ± 467.0 (1860)	0.944	3088.4 ± 481.93 (2201)	0.872
Delivery place, % institution (*n*)	83.9% (334)	88.3% (2080)	0.023	89.3% (2272)	0.004
Mode of delivery, % cesarean section (*n*)	4.8% (19)	6.9% (162)	0.729	6.8% (177)	0.066

^1^SHINE, Sanitation Hygiene Infant Nutrition Efficacy study; Z-DHS, Zimbabwe Demographic and Health Survey.

^2^The 2015 Z-DHS assessed maternal attitude toward intimate partner violence with this question: “In your opinion, is a husband justified in hitting or beating his wife in the following situations: if she goes out without telling him; neglects the children; argues with him; refuses to have sex with him; burns the food; commits infidelity?” The SHINE survey assessed this by asking women to give a response (strongly agree, agree, neutral, disagree, strongly disagree) to this statement: “A woman must accept that her husband or partner beats her, in order to keep the family together.” In the merged data set, we created a dichotomous variable, classifying a mother as believing domestic violence may be justifiable if she replied “yes” to any of the 6 circumstances in Z-DHS or if she replied “strongly agree” or “agree” in SHINE.

To identify maternal and infant characteristics associated with nonoptimal breastfeeding practice, we modeled the association between receipt of each intervention module and each outcome (late initiation, early mixed feeding, and late mixed feeding) using the merged data set. Models included variables that were associated with the breastfeeding outcome within the SHINE data set. We used backward elimination stepwise regression modeling, forcing receipt of EBF module variables to stay in the model, to construct parsimonious models (*P* > 0.2 to remove and <0.05 to add). We tested retained variables for statistical interactions with receipt of the intervention module.

Within the SHINE trial, a range of household and individual characteristics were measured, which we hypothesized would explain heterogeneity along the program impact pathways linking implementation of the randomized interventions with child health outcomes (12). In post hoc analyses, we investigated whether the following factors, plausibly related to early infant feeding behaviors, modified the effect of the breastfeeding intervention on EI or EBF prevalence: mode and place of delivery and infant birth weight and sex. We also tested a variable for whether the mother believed husbands beating their wives was justifiable; this belief was common in both surveys, and we hypothesized that inequitable gender norms may reduce a mother's ability to respond to new information. All analyses were conducted using Stata statistical software version 14.1 (Stata Corporation).

## Results

A total of 2442 and 2728 infants were available for follow-up in their homestead at the 1 and 3 mo visits, respectively. Follow-up was low at these visits primarily because of the cultural practice for women (especially primiparious) to return to their parental home during the perinatal period (**Supplemental Figure 1**). Mothers who provided data were, on average, 1.5 y older and had a higher parity (reflecting the departure of primiparous women) than women who did not provide data, but no other meaningful differences were observed (**Supplemental Tables 1** and **2**).

The majority of mothers in the SHINE and Z-DHS data sets had completed primary school and were married, Christian, not formally employed, and living in homes without electricity or piped water (Table 1). The HIV prevalence in both surveys was similar to the national prevalence of 15% (6). Variables that differed significantly across the surveys were considered when building the multivariable models as described in the methods.

**FIGURE 1:**
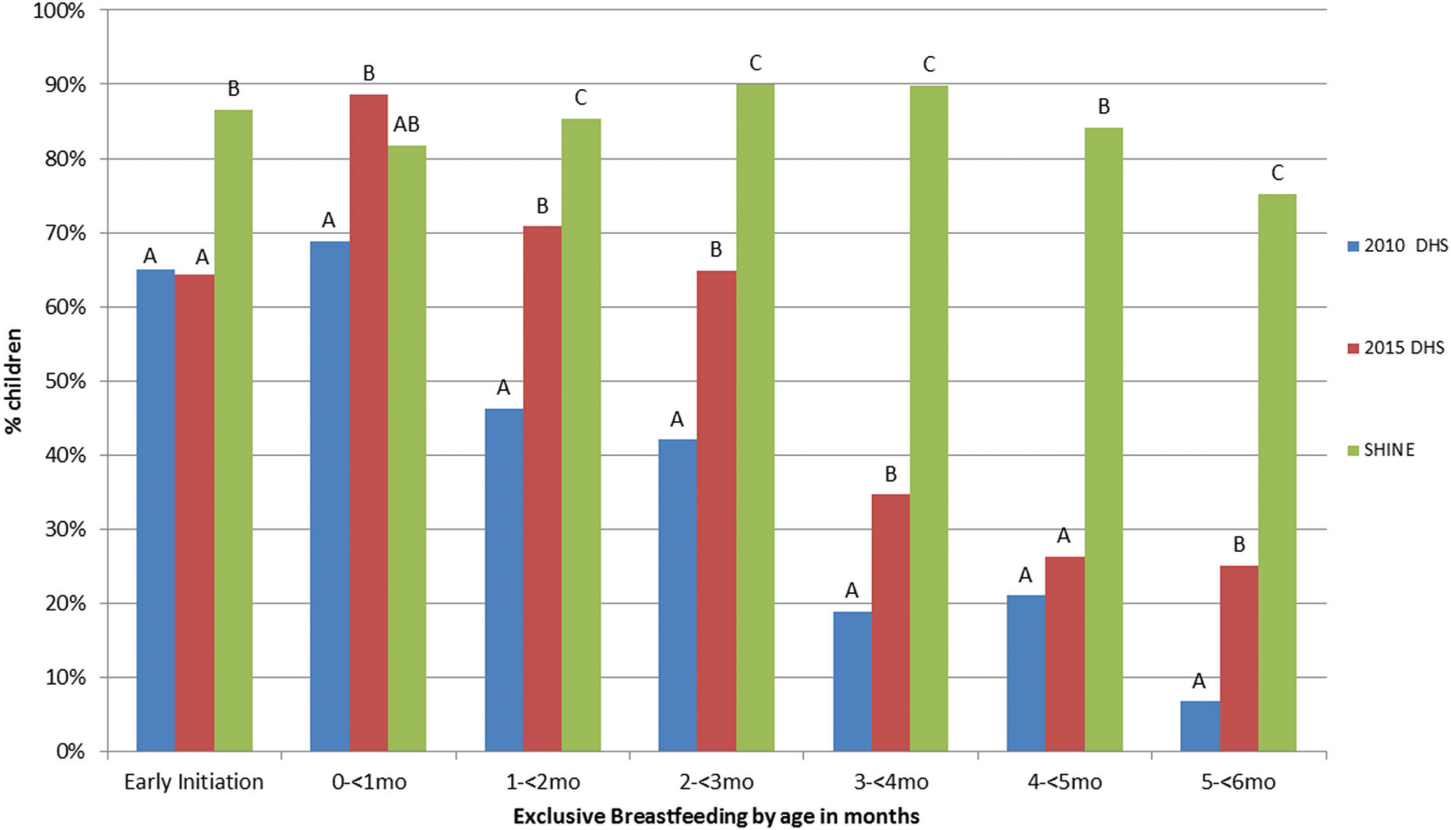
Prevalence of early initiation of breastfeeding and exclusive breastfeeding according to months of age for mothers in SHINE compared with rural mothers participating in the 2010 and 2015 Zimbabwe Demographic Health Surveys. Sample sizes for 0 to <1, 1 to <2, 2 to <3, 3 to <4, 4 to <5, and 5 to <6 mo are respectively: 32, 95, 81, 90, 100, and 89 for DHS 2010; 62, 72, 71, 78, 65, and 36 for DHS 2015; and 277, 1410, 802, 1474, 343, and 176 for SHINE. No significant difference by age group is indicated by the same letter. DHS, Demographic Health Survey; SHINE, Sanitation Hygiene Infant Nutrition Efficacy study.

EI prevalence was 86.6% in SHINE participants compared with 64.3% in the rural Z-DHS (**Figure 1**, *P* < 0.001).[Table tbl2] EBF prevalence did not differ between SHINE and rural Z-DHS infants in the first month after birth, when more than 80% of infants in both surveys were EBF. Thereafter, prevalence of EBF steadily declined with infant age among Z-DHS infants, but persisted at high prevalence in SHINE infants (Figure 1; for historical reference, EI and EBF prevalence reported in the 2010 Z-DHS are also shown).

Using the merged data set, the unadjusted relative risks (95% CI) of late initiation, early mixed feeding (0–12 wk), and late mixed feeding (12–25 wk) were 0.37 (0.31, 0.44), 0.55 (0.42, 0.71), and 0.17 (0.15, 0.20), respectively, among women enrolled in SHINE compared with those in 2015 Z-DHS; these relative risks changed little after adjusting for potential confounders (**Table 2**).

**TABLE 2 tbl2:** Unadjusted and adjusted associations of SHINE breastfeeding intervention exposures on breastfeeding outcomes, using the rural 2015 Zimbabwe Demographic Health Survey as comparison^1^

	Late initiation (95% CI)		Early mixed feeding (95% CI)		Late mixed feeding (95% CI)	
SHINE breastfeeding intervention exposure	Unadjusted IRR	Adjusted IRR	Unadjusted IRR	Adjusted IRR	Unadjusted IRR	Adjusted IRR
Enrolled in SHINE	0.37 (0.31, 0.44)	0.35 (0.27, 0.44)	0.55 (0.42–0.71)	0.45 (0.32–0.65)	0.17 (0.15–0.20)	0.21 (0.17–0.26)
Received 1st lesson, 7-mo pregnancy	0.46 (0.39, 0.55)	0.47 (0.37, 0.60)	0.60 (0.48–0.74)	0.59 (0.44–0.80)	0.25 (0.22–0.30)	0.33 (0.27–0.41)
Received 2nd lesson, 1 w postpartum			0.72 (0.57–0.90)	0.65 (0.49–0.88)	0.23 (0.20–0.27)	0.30 (0.25–0.37)
Received 3rd lesson, 1 mo postpartum			0.62 (0.49–0.78)	0.56 (0.41–0.76)	0.22 (0.19–0.25)	0.29 (0.24–0.36)
Received 4th lesson, 3 mo postpartum					0.21 (0.18–0.24)	0.28 (0.23–0.35)

^1^Models were adjusted for potential confounders as described in the methods. These differed by outcome. For late initiation: institutional delivery, marital status, believes intimate partner violence may be justifiable, HIV status, infant sex. For early mixed feeding: marital status, believes intimate partner violence may be justifiable, maternal height, infant birth weight, sex, and infant age at survey. For late mixed feeding: believes intimate partner violence may be justifiable, maternal age, schooling and HIV status, infant birth weight, sex, and infant age at survey. All variables were retained in the final model regardless of statistical significance. IRR, incident rate ratio; SHINE, Sanitation Hygiene Infant Nutrition Efficacy study.

Women who received Module 1 had a 28% (95% CI: 11%, 41%) lower risk of late breastfeeding initiation compared with women who did not receive the module; this estimate changed little after adjustment for potential confounders (Table 2). Receipt of each of the first 3 EBF Modules was associated with ∼30–40% lower risk of early mixed feeding; these estimates changed little with adjustment (Table 2). Finally, receipt of each of the 4 modules was associated with ∼80% lower risk of late mixed feeding, which attenuated to ∼70% after adjustment (Table 2).

**FIGURE 2:**
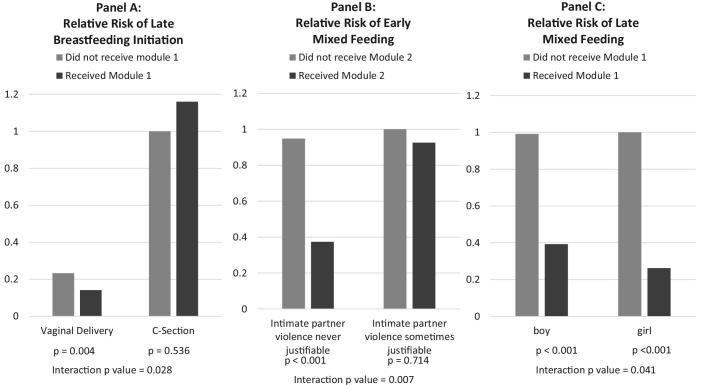
Illustrations of effect modification of the benefit of the SHINE breastfeeding intervention on breastfeeding outcomes. (A) Relative risk of late initiation of breastfeeding by cesarean delivery and receipt of Breastfeeding Module 1 in late pregnancy. Results were adjusted for place of delivery, mother's belief that intimate partner violence was sometimes justifiable, mother's HIV status, and infant sex. (B) Relative risk of early mixed feeding (1–2.5 mo) by belief that intimate partner violence was sometimes justifiable and receipt of Breastfeeding Module 2 in week 1 postpartum. Results were adjusted for mother's marital status and height, and infant's birth weight, age at survey, and sex. (C) Relative risk of late mixed feeding (2.5–5.9 mo) by infant sex and receipt of Breastfeeding Module 1 in late pregnancy. Results were adjusted for belief that intimate partner violence was sometimes justifiable, mother's age, education and HIV status, and infant's birth weight and age at survey. See Table 3 for a summary of all interactions observed. SHINE, Sanitation Hygiene Infant Nutrition Efficacy study.

Birth weight did not modify the effect of the intervention on EI or EBF prevalence. However, delivery characteristics were strongly related to EI. In parsimonious models, women who delivered outside a health institution were 1.96 (95% CI: 1.38, 2.78) (*P* < 0.001) times more likely to initiate breastfeeding after the first hour of life. However, the 7% of women who delivered by cesarean section were 6.24 (95% CI: 4.97, 7.83) (*P* < 0.001) times more likely to initiate breastfeeding late, and receipt of Module 1 was not associated with EI prevalence for these women (*P* for interaction term = 0.028; **Figure 2****A**). Delivery characteristics were not related to early or late EBF practices.

The relative risk (95% CI) of early and late mixed feeding was, respectively, 2.07 (1.59, 2.68) (*P* < 0.001), and 1.37 (1.11, 1.67) (*P* = 0.003) among women who believed that intimate partner violence may be justifiable compared with women who did not. This belief modified the effect of modules 1, 2, and 3 on early mixed feeding (**Table 3**). Among women who believed intimate partner violence may be justifiable, none of the modules had a significant effect on early mixed feeding, whereas all 3 modules were associated with large reductions in early mixed feeding among women who did not hold this belief (Figure 2B). Importantly, 41% and 61% of women in the Z-DHS and SHINE, respectively, held this belief, suggesting that the intervention would have little effect on early mixed feeding among about half the women in Zimbabwe.

**TABLE 3:** Summary of factors that negatively modified the effect of the Sanitation Hygiene Infant Nutrition Efficacy study breastfeeding modules on breastfeeding outcomes^1^

	Significant effect modifiers of breastfeeding outcomes		
Intervention module (see Box 1)	Late initiation	Early mixed feeding	Late mixed feeding
1	Cesarean section^2^	Intimate partner violence justifiable^3^	Infant is male^1^^,^^4^
2		Intimate partner violence justifiable^1^^,^^5^	Intimate partner violence justifiable^6^
3		Intimate partner violence justifiable^7^	None
4			Infant is male^8^

^1^See Figure 2 for a more detailed presentation of these interactions. All interactions reflect negative effect modification, meaning that the intervention was less effective in women with these characteristics.

^2^Interaction *P* < 0.001; model adjusted for institutional delivery; maternal attitude toward intimate partner violence, marital status, height, age, and HIV status; infant birth weight and sex.

^3^Interaction *P* = 0.048; model adjusted for maternal marital status and height; infant birth weight, age, and sex.

^4^Interaction *P* = 0.036; model adjusted for maternal attitude intimate partner violence, age, schooling, HIV status; and infant birth weight and age.

^5^Interaction *P* = 0.009; model adjusted for maternal marital status and height; infant birth weight, age, and sex.

^6^Interaction *P* = 0.008; model adjusted for maternal age, schooling, and HIV status; infant birthweight, age, and sex.

^7^Interaction *P* = 0.035; model adjusted for maternal marital status and height; infant birth weight, age, and sex.

^8^Interaction *P* = 0.039; model adjusted for maternal age, schooling, and HIV status; infant birth weight and age.

Male infants were 1.26 (0.99, 1.60) (*P* = 0.06) and 1.31 (1.06, 1.60) (*P* = 0.011) times more likely than females to be early and late mixed fed, respectively. Moreover, the modules were less effective in reducing late mixed feeding of boys. This interaction was statistically significant for modules 1 and 4 (Table 3, Figure 2C).

## Discussion

The EI and EBF prevalence observed among SHINE women following implementation of this breastfeeding intervention are among the highest reported by research studies testing breastfeeding promotion interventions (15). We speculate that 3 aspects of the intervention were key to its impact. First, the educational messages focused on culturally relevant, time-specific barriers to optimal practices. Common breastfeeding promotion messages that were not used in this study (e.g., “breast milk is the best food for your baby”) do not address the specific beliefs of Zimbabwean mothers that an open fontanel or colic should be treated or prevented by oral remedies. Second, the intervention messages were delivered at moments when the information was most relevant to maternal decisions. Third, the VHWs were trained, resourced, and supervised to implement the intervention with a high degree of excellence. Thus, although implementation reached thousands of households and was carried out by government-employed VHWs, this was not a pure effectiveness evaluation because VHWs were resourced at a higher level than is usual outside a research setting. Important work remains to adapt the SHINE materials and experience for larger scale-up.

Our analyses point to 3 ways in which the intervention could be improved:

- First, promote institutional deliveries. Mothers delivering within a health institution were more likely to initiate breastfeeding early. Encouragingly, between 2011 and 2015, the proportion of births attended by skilled health staff in Zimbabwe increased from 66% to 78% (16).
- Second, provide special support for mothers delivering by cesarean section. As widely reported (17) cesarean section is a risk factor for late breastfeeding initiation. This issue is relatively minor for Zimbabwe, 1 of only 3 countries in the world where cesarean delivery prevalence did *not* increase between 1990 and 2014, but remained low at 6% (18). Other regions have experienced large increases over this period (18). Our findings suggest that limiting cesarean sections to those medically required and providing specialized breastfeeding support for women following cesarean section will increase EI prevalence. WHO has stated that breastfeeding can often be initiated immediately after delivery by cesarean section for the majority of women (19).
- Third, address gender norms. Women who believed that intimate partner violence may be justifiable were less likely to practice EBF and less responsive to the intervention. Intimate partner violence affects 1 in 3 women worldwide (20). In prior analyses, gender norm attitudes consistent with lower women's empowerment were associated with more depressive symptoms, less social support, and more time stress (21). Thus, these women may lack the capacity to act on new education. Fortunately, there is growing evidence of the effectiveness of social interventions to prevent violence against women (22–24). Our findings suggest that such interventions will not only directly benefit women, but also improve care for their children.

Mothers were less likely to practice EBF in male infants, and the intervention was less effective among mothers of boys than girls. We hypothesize, consistent with ethnographic assessments elsewhere (25), that boys are perceived to have a bigger appetite or require the perceived strengthening effects of nonbreastmilk foods, compared with girls. A message that breast milk is “strong food, even for boys,” should be tested before future iterations of the intervention.

Our study has several limitations. First, Z-DHS data were cross-sectional, whereas SHINE data were longitudinal. We do not think this affects our inferences because comparisons were made cross-sectionally. Second, our evaluation did not use a randomized design; however, we used concurrent comparisons and statistical controls to estimate the effect of the intervention within CIs. Third, SHINE mothers who provided data at 1 and 3 mo were older than those who did not. Within the SHINE data set, maternal age was not associated with EI, EBF at 1 mo, or still breastfeeding at 6 mo, but there was a trend toward older women being more likely to carry out EBF at 3 mo than younger women (data not shown). Although the magnitude of this effect was small and borderline significant, this finding indicates that we may have overestimated the impact our intervention would have had among a younger population of women. Finally, data regarding breastfeeding practices relied on maternal recall, and there may have been greater courtesy bias in SHINE than Z-DHS. We minimized this bias by having different cadres implement the intervention and measure the breastfeeding practices. There may also have been differential recall bias for the EI outcome: Z-DHS assessed EI from mothers of children 0 to <24 mo of age, whereas SHINE children were all <12 wk of age.

## Conclusions

In summary, our analyses indicate that this intervention was highly effective in increasing prevalence of EI and EBF. Future work in Zimbabwe should focus on delivering it at scale, increasing institutional deliveries, providing more support for women following cesarean section delivery, and addressing inequitable gender attitudes. Formative studies to identify context and infant age-specific barriers to EI and EBF may inform improvement of breastfeeding practices elsewhere.

## Supplementary Material

Supplemental Files

## Notes

This study was funded by the Bill and Melinda Gates Foundation (OPP1021542 and OPP1143707); United Kingdom Department for International Development (DFID/UKAID); Wellcome Trust (093768/Z/10/Z and 108065/Z/15/Z); and Swiss Agency for Development and Cooperation. The funders approved the study design but had no role in the data collection, data analysis, data interpretation, or writing of the report.

Members of the SHINE Trial Team not named in this paper are listed in https://doi.org/10.1093/cid/civ844.

The datasets used and/or analyzed during the current study are available from the corresponding author on request.

Supplemental Tables 1 and 2 and Supplemental Figure 1 are available from the “Supplementary data” link in the online posting of the article and from the same link in the online table of contents at https://academic.oup.com/cdn/.

Present address for MNNM: Global Alliance for Improved Nutrition, GAIN NoSCA, LLC1509 16th St NW, FL 7 Washington, DC 20036, USA.

Present address for RJS: Office of the President, Goshen College, 1700 S Main St, Goshen, IN 46526, USA.

Abbreviations used: EBF, exclusive breastfeeding; EI, early initiation; SHINE, Sanitation Hygiene Infant Nutrition Efficacy study; VHW, village health worker; Z-DHS, Zimbabwe Demographic and Health Survey.
