# Supplementary material for: Early Initiation and Exclusivity of Breastfeeding in Rural Zimbabwe: Impact of a Breastfeeding Intervention Delivered by Village Health Workers
Source: Curr Dev Nutr. 2019 Feb 28;3(4):nzy092. doi: 10.1093/cdn/nzy092 (PMC6438822; doi:10.1093/cdn/nzy092)
Supplement: Supplemental Files [file nzy092_supplemental_files.zip › Online Supplemental tables and Figure.docx]

**Early initiation and exclusivity of breastfeeding in rural Zimbabwe: impact of a breastfeeding intervention delivered by village health workers**

Mduduzi N.N. Mbuya, Cynthia R. Matare, Naume V. Tavengwa, Bernard Chasekwa, Robert Ntozini, Florence D. Majo, Ancikaria Chigumira, Cynthia M.Z. Chasokela, Andrew J. Prendergast, Lawrence H. Moulton, Rebecca J. Stoltzfus, and Jean H. Humphrey for The SHINE Trial Team.

**Supplementary Table 1. Baseline characteristics of SHINE mothers and infants who provided and did not provide data at the 1-month visit.**

| **Baseline characteristic** | **Provided data at 1 month visit** | **Did not provide data at 1 month visit** | **P value** |
| --- | --- | --- | --- |
|  | (N=2413 mothers and 2442 infants) | (N=2289 mother and 2208 infants) |  |
|  |  |  |  |
| **Maternal characteristics** |  |  |  |
| Age(y), mean(SD) [n/N] | 26.9 (6.66) [2235] | 25.4 (6.52) [1831] | <0.001 |
| Height (cm), mean (SD[n/N] | 160.2 (5.92) [2351] | 160.1 (5.88) [2075] | 0.741 |
| Completed schooling (y), median(IQR)[n/N] | 10 (9,11) [2308] | 10 (9,11) [2015] | 0.149 |
| Parity, median (IQR[n/N] | 2 (1,3) [1710] | 2 (1,3) [1420] | <0.001 |
| Marital status, %married [n/N] | 95.4% [2188/2293] | 94.8% [1895/1999] | 0.374 |
| Believes that being beaten by an intimate partner is  sometimes justifiable, %yes[n/N] | 61.4% [1415/2305] | 74.5% [1419/1905] | <0.001 |
| Employed, %yes [n/N] | 8.54% [197/2307] | 8.8% [168/1907] | 0.341 |
| Religion: |  |  |  |
| Apostolic, %(n/N) | 47.2% [1091/2310] | 46.7% [940/2014] |  |
| Other Christian, %(n/N) | 44.3% [1024/2310] | 45.1% [909/2014] | 0.872 |
| Other religion, %(n/N) | 8.4% [195/2310] | 8.2% [165/2014] |  |
| **Household characteristics** |  |  |  |
| Electricity, % yes (n/N) | 2.5% [57/2311] | 3.2% [61/1904] | 0.345 |
| Any latrine, %yes [n/N] | 39.9% [907/2272] | 39.9% [755/1888] | 0.964 |
| Improved floor, %yes [n/N] | 54.6% [1245/2281] | 53.8% [1012/1882] | 0.602 |
| Improved roof, % yes [n/N] | 47% [1133/2413] | 43.6% [949/2179] | <0.001 |
| Time to fetch drinking water(min), median (IQR)[n/N] | 10 (5,20) [2274] | 10 (5,20) [1907] | 0.181 |
| Wealth Quintile: |  |  |  |
| Lowest, %(n/N) | 18.2% (421/2313 ] | 21.3% [405/1903] |  |
| Second, %(n/N) | 19.8% [457/2313 ] | 20.3% [390/1903] |  |
| Middle, %(n/N) | 21.7% [501/2313 ] | 18.4% [350/1903] | 0.122 |
| Fourth, %(n/N) | 21.0% [487/2313 ] | 19.1% [364/1903] |  |
| Highest, %(n/N) | 19.3% [447/2313 ] | 20.7% [397/1903] |  |
| Household size, median (IQR[n/N] | 5 (3,6) [2329] | 5 (3,6) [2025] | 0.635 |
| **Infant characteristics** |  |  |  |
| Gender, %female [n/N] | 49.8% [1216/2442] | 49.8% [1089/2189] | 0.975 |
| Birth weight (g), mean (SD[n/N] | 3.09 (.47) [1868] | 3.08 (.49) [1711] | 0.751 |
| Delivery place, %institution [n/N] | 88.2% [2079/2356] | 88.4% [1586/1794] | 0.871 |
| Mode of delivery, %caesarean section [n/N] | 6.88% [162/2353] | 6.5% [121/1867] | 0.662 |

**Supplementary Table 2. Baseline characteristics of SHINE mothers and infants who provided and did not provide data at the 3-month visit.**

| **Baseline characteristic** | **Provided data at 3 month visit** | **Did not provide data at 3 month visit** | **P value** |
| --- | --- | --- | --- |
|  | (N=2695 mothers and 2728 infants) | (N=1881 mothers and 1903 infants) |  |
| **Maternal characteristics** |  |  |  |
| Age(y), mean (SD[n/N] | 26.7 (6.68) [2490] | 25.5 (6.48) [1560] | <0.001 |
| Height(cm), mean (SD[n/N] | 160.2(5.88) [2627] | 160.1 (5.95) [1782] | 0.509 |
| Completed schooling (y), median (IQR)[n/N] | 10 (9,11) [2587] | 10 (8,11) [1720] | 0.038 |
| Parity, median (IQR[n/N] | 2 (1,3) [2034] | 2 (1,3) [1090] | 0.003 |
| Marital status, %married [n/N] | 96% [2466/2569] | 93.9% [1603/1707] | 0.009 |
| Believes that being beaten by an intimate partner is  sometimes justifiable, %yes [n/N] | 61.5% [1575/2562] | 76.5% [1247/1631] | <0.001 |
| Employed, %yes [n/N] | 8.9% [230/2563] | 8.1% [133/1634] | 0.654 |
| Religion: |  |  |  |
| Apostolic, %(n/N) | 45.9% [1188/2589] | 48.7% [837/1719] | <0.001 |
| Other Christian, % (n/N) | 47.2% [1222/2589] | 40.8% [702/1719] |  |
| Other religion, %(n/N) | 6.9% [179/2589] | 10.5% [180/1719] |  |
| **Household characteristics** |  |  |  |
| Electricity, % yes (n/N) | 2.7% [68/2565] | 3.1% [50/1633] | 0.207 |
| Any latrine, %yes [n/N] | 41.6% [1051/2528] | 37.5% [605/1615] | 0.008 |
| Improved floor, %yes [n/N] | 55.9% [1414/2530] | 51.5% [832/1616] | 0.006 |
| Improved roof, % yes [n/N] | 48.4% [1305/2695] | 40.8% [766/1878] | <0.001 |
| Time to fetch drinking water(min), median (IQR)[n/N] | 10 (5,20) [2532] | 10 (5,20) [1613] | 0.038 |
| Wealth Quintile: |  |  |  |
| Lowest, %(n/N) | 18.29% [469/2564] | 21.53% [352/1635] | 0.019 |
| Second, %(n/N) | 20.44% [524/2564] | 19.51% [319/1635] |  |
| Middle, %(n/N) | 20.48% [525/2564] | 19.76% [323/1635] |  |
| Fourth, %(n/N) | 20.01% [513/2564] | 20.37% [333/1635] |  |
| Highest, %(n/N) | 20.79% [533/2564] | 18.84% [308/1635] |  |
| Household size, median (IQR[n/N] | 4 (3,6) [2607] | 5 (3,6) [1731] | 0.114 |
| **Infant characteristics** |  |  |  |
| Gender, %female [n/N] | 49.6% [1354/2728] | 50% [941/1884] | 0.834 |
| Birth weight(g), mean (SD[n/N] | 3.1 (.48) [2210] | 3.1(.47) [1365] | 0.435 |
| Delivery place, %institution [n/N] | 89.4% [2273/2544] | 86.8% [1384/1595] | 0.012 |
| Mode of delivery, % caesarean section [n/N] | 6.8% [177/2586] | 6.4% [104/1623] | 0.321 |





Supplementary Figure 1. Flow of participants
