# Supplementary material for: Early Initiation and Exclusivity of Breastfeeding in Rural Zimbabwe: Impact of a Breastfeeding Intervention Delivered by Village Health Workers
Source: Curr Dev Nutr. 2019 Feb 28;3(4):nzy092. doi: 10.1093/cdn/nzy092 (PMC6438822; doi:10.1093/cdn/nzy092)
Supplement: Supplemental Files [file nzy092_supplemental_files.zip › STROBE_checklist_cdn.docx]

STROBE Statement—checklist of items that should be included in reports of observational studies

|  | Item No. | Recommendation | Page  No. | Relevant text from manuscript |
| --- | --- | --- | --- | --- |
| **Title and abstract** | 1 | (*a*) Indicate the study’s design with a commonly used term in the title or the abstract | 3 (abstract) | In a cross-sectional analysis, EI rates were….. |
|  |  | (*b*) Provide in the abstract an informative and balanced summary of what was done and what was found | 3 | We compared EI and EBF rates among women who received the SHINE intervention with women in the 2015 –DHS.  Results section reports observed rates from both surveys with absolute differences (95CI) |
| Introduction | | | |  |
| Background/rationale | 2 | Explain the scientific background and rationale for the investigation being reported | 5 | Optimal breastfeeding includes…  In Zimbabwe…suboptimal breastfeeding was the leading risk factor for disease burden…  In 2011 we conducted mixed methods formative research… We found (the barriers). Based on these an intervention was designed… … |
| Objectives | 3 | State specific objectives, including any prespecified hypotheses | 6 | We aimed to assess the effectiveness of the …intervention… and identify …characteristics associated with poor uptake |
| Methods | | | |  |
| Study design | 4 | Present key elements of study design early in the paper | 7 | Larger SHINE trial described, breastfeeding intervention described, measurement of outcomes in each survey described. |
| Setting | 5 | Describe the setting, locations, and relevant dates, including periods of recruitment, exposure, follow-up, and data collection | 7  9 | The study area was the rural population of two district in central Zimbabwe…. Between November 2012 through March 2015; the breastfeeding intervention included four modules delivered at four time-points..  Within SHINE, nurses assessed breastfeeding practices…(assessment tools described) Within the 2015 Z-DHS, EI was assessed…EBF was assessed… |
| Participants | 6 | (*a*) *Cohort study*—Give the eligibility criteria, and the sources and methods of selection of participants. Describe methods of follow-up  *Case-control study*—Give the eligibility criteria, and the sources and methods of case ascertainment and control selection. Give the rationale for the choice of cases and controls  *Cross-sectional study*—Give the eligibility criteria, and the sources and methods of selection of participants | 7 | Women were eligible if they were permanent residents in the rural areas of the study area, confirmed pregnant by a positive pregnancy test and met the gestational age cut-off in force at the time of their recruitment. |
|  |  | (*b*) *Cohort study*—For matched studies, give matching criteria and number of exposed and unexposed  *Case-control study*—For matched studies, give matching criteria and the number of controls per case |  |  |
| Variables | 7 | Clearly define all outcomes, exposures, predictors, potential confounders, and effect modifiers. Give diagnostic criteria, if applicable | 9 | SHINE: EI was assessed… responses < 1 hour were classified as EI. EBF was assessed using a tool… Children were classified as EBF if…  DHS: EI was assessed…responses < 1 h were classified as EI. EBF was assessed… children were classified as EBF if…  We define ‘Early EBF’as…and ‘Late EBF’ as…  We estimated risks and risk factors of …..‘late initiaion’… ‘early mixed feeding’,… and ‘late mixed feeding’ |
| Data sources/ measurement | 8* | For each variable of interest, give sources of data and details of methods of assessment (measurement). Describe comparability of assessment methods if there is more than one group | 7-9  9 | Design and methods of SHINE trial…  The lists of foods used to assess breastfeeding exclusivity in SHINE and Z-DHS…  The age distributions…differed between the two surveys |
| Bias | 9 | Describe any efforts to address potential sources of bias | 9  10 | The lists of foods used…were not exactly alike…However…  To improve comparability… |
| Study size | 10 | Explain how the study size was arrived at | 12 | Of the women enrolled in SHINE, 2442 and 2728 were available…For DHS study sample size is described in detail in cited reference. |

Continued on next page

| Quantitative variables | 11 | Explain how quantitative variables were handled in the analyses. If applicable, describe which groupings were chosen and why | 10 | We defined intervention exposure in two ways… we created a dichotomous variable for (receipt of) each modules |
| --- | --- | --- | --- | --- |
| Statistical methods | 12 | (*a*) Describe all statistical methods, including those used to control for confounding | 10-12 | We compared proportions  We calculated unadjusted RR (95% CI)  We constructed multivariate Poisson regressions models with a log link  To select covariates in multivariate regression analyses we…  We tested these association within the SHINE set, for associations significant at p<0.2, we tested whether the distribution… differed… to identify potential confounders  Both studies…constructed asset indices as an indicator of wealth  To identify …characteristics associated with non-optimal breastfeeding practice…  We used backward elimination stepwise regression modelling…to construct parsimonious models  We tested retained variables for statistical interactions with receipt of the intervention model. |
|  |  | (*b*) Describe any methods used to examine subgroups and interactions | 12 | We tested retained variables for statistical interactions with receipt of the intervention model. |
|  |  | (*c*) Explain how missing data were addressed | 12 and Supplementary Figure 1. Flow of participants |  |
|  |  | (*d*) *Cohort study*—If applicable, explain how loss to follow-up was addressed  *Case-control study*—If applicable, explain how matching of cases and controls was addressed  *Cross-sectional study*—If applicable, describe analytical methods taking account of sampling strategy | Not relevant |  |
|  |  | (*e*) Describe any sensitivity analyses | Not relevant |  |
| Results | | | | |
| Participants | 13* | (a) Report numbers of individuals at each stage of study—eg numbers potentially eligible, examined for eligibility, confirmed eligible, included in the study, completing follow-up, and analysed | Supplementary Figure 1. Flow of participants |  |
|  |  | (b) Give reasons for non-participation at each stage | Supplementary Figure 1. Flow of participants |  |
|  |  | (c) Consider use of a flow diagram | Supplementary Figure 1. Flow of participants |  |
| Descriptive data | 14* | (a) Give characteristics of study participants (eg demographic, clinical, social) and information on exposures and potential confounders | 21, Table 1 (baseline factors of all data sets) |  |
|  |  | (b) Indicate number of participants with missing data for each variable of interest | 21 | n’s presented for all values |
|  |  | (c) *Cohort study*—Summarise follow-up time (eg, average and total amount) |  |  |
| Outcome data | 15* | *Cohort study*—Report numbers of outcome events or summary measures over time |  |  |
|  |  | *Case-control study—*Report numbers in each exposure category, or summary measures of exposure |  |  |
|  |  | *Cross-sectional study—*Report numbers of outcome events or summary measures | Figure 1 |  |
| Main results | 16 | (*a*) Give unadjusted estimates and, if applicable, confounder-adjusted estimates and their precision (eg, 95% confidence interval). Make clear which confounders were adjusted for and why they were included | Table 2 | Unadjusted and adjusted RR presented; footnote includes all covariates in adjusted models |
|  |  | (*b*) Report category boundaries when continuous variables were categorized | 10 | Infant age boundaries defined for Early EBF, Late EBF, Late initiation, early mixed feeding and late mixed feeding |
|  |  | (*c*) If relevant, consider translating estimates of relative risk into absolute risk for a meaningful time period | 3 | Absolute differences (95% CI) presented for primary outcomes |

Continued on next page

| Other analyses | 17 | Report other analyses done—eg analyses of subgroups and interactions, and sensitivity analyses | 13-14 | …Women who delivered outside a health institution…Women who delivered by Caesarian section, Women who believed intimate partner violence may be justifiable…Male infants were more likely…than females |
| --- | --- | --- | --- | --- |
| Discussion | | | | |
| Key results | 18 | Summarise key results with reference to study objectives | 14  15 | EI and EBF rates observed…are among the highest reported  Our analyses point to three ways in which the intervention could be improved… |
| Limitations | 19 | Discuss limitations of the study, taking into account sources of potential bias or imprecision. Discuss both direction and magnitude of any potential bias | 16 | Our study has several limitations… |
| Interpretation | 20 | Give a cautious overall interpretation of results considering objectives, limitations, multiplicity of analyses, results from similar studies, and other relevant evidence | 14  15 | We speculate that three aspects of the intervention were key to its impact  Our analyses point to three ways in which the intervention could be improved |
| Generalisability | 21 | Discuss the generalisability (external validity) of the study results | 14 and 16 | We state that an element of the intervention which likely led to its success is that it was context-specific (ie, our intervention would not be expected to be effective in other contexts) but also that conducting formative studies to identify context and infant age-specific barriers to EI and EBF may inform improvement of breastfeeding practices elsewhere (ie, our method is likely to be generalisable) |
| Other information | |  | | |
| Funding | 22 | Give the source of funding and the role of the funders for the present study and, if applicable, for the original study on which the present article is based | 2  7 | Source of funding and role of funders stated.  The design and methods of the SHINE trial have been previously reported (reference #8) |

*Give information separately for cases and controls in case-control studies and, if applicable, for exposed and unexposed groups in cohort and cross-sectional studies.

**Note:** An Explanation and Elaboration article discusses each checklist item and gives methodological background and published examples of transparent reporting. The STROBE checklist is best used in conjunction with this article (freely available on the Web sites of PLoS Medicine at http://www.plosmedicine.org/, Annals of Internal Medicine at http://www.annals.org/, and Epidemiology at http://www.epidem.com/). Information on the STROBE Initiative is available at www.strobe-statement.org.
